# Advances in the Gene–Diet Interactions and Human Health

**DOI:** 10.3390/nu18101577

**Published:** 2026-05-15

**Authors:** Lusânia Maria Greggi Antunes

**Affiliations:** School of Pharmaceutical Sciences of Ribeirao Preto, University of Sao Paulo, Sao Paulo 14043-900, Brazil; lusania@fcfrp.usp.br

This editorial is for the special volume “Advances in the Gene–Diet Interactions and Human Health”, which addresses the applications and importance of Nutrigenomics in promoting health and gene–diet interactions in the prevention of human diseases.

One important area of science is the field of nutrigenomics, that is, the effects of diet on gene expression, and the extent of genetic differences between individuals that influence responses to specific dietary patterns, functional foods, or nutraceuticals [1,2]. The interaction between genotype and diet in modulating risks is an exciting area of research with respect to the effects of micronutrients on DNA. Nutrigenomics can also be defined as the interaction between nutraceuticals in the diet and an individual’s genome’s response to different diets [3,4]. The application of molecular biology techniques allows the simultaneous investigation of genetic polymorphisms associated with dietary diseases (nutrigenetics), micronutrients that induce changes in DNA methylation and chromosome chromatin (nutritional epigenomics), and nutrients that induce changes in gene expression (nutritional transcriptomics) [5]. The combined efforts of industry, government, and academia are essential in developing a comprehensive and integrated strategy for research on nutraceuticals and functional foods in health promotion, with the possible implementation of a new complementary preventive medicine based on nutritional intervention. The success of this area depends on the dedication of scientists who use molecular biology techniques (omics) to identify the cellular and molecular targets of nutraceuticals and how these targets may be related to disease prevention. Identifying these mechanisms can be a major challenge, especially when considering the multiple pathways of interactions between individual food components and the influence of genetic susceptibilities [6]. Therefore, there is ample justification for creating an innovative research model to explore the role of diet in health promotion and disease prevention.

This special volume features nine articles with different approaches to gene–diet interaction, whose results are important for a better understanding of the interaction mechanisms between diet, genes, and health promotion, encouraging the reading of current and relevant articles with new knowledge in the field of gene–diet interactions.

Zand et al. (Contribution 1) investigated the epigenetic effects of tartrazine, a synthetic azo dye used in pharmaceuticals, cosmetics, and food, on human tumor cell lines. The authors concluded that tartrazine induced DNA damage in human cells and may play a role in the mechanisms of carcinogenesis and epigenetic alterations. Matsuda et al. (Contribution 2) conducted a comprehensive review on the role of epigenetic alterations of N6-methyladenine (m6A) in the regulation of various diseases, particularly metabolic dysfunction-associated steatotic liver disease (MASLD). The review emphasizes the impact of mitochondrial dysfunction and the development of therapeutic strategies for MASLD.

Two articles addressed gene–diet interactions with the use of nutraceuticals in intervention strategies in the carcinogenesis process. Wu et al. (Contribution 3) evaluated the effects of the nutraceuticals sulforaphane, an antioxidant isothiocyanate, and inulin, as a dietary fiber, in the prevention of breast cancer in transgenic estrogen-representing receptor-negative mice. The combined treatment with nutraceuticals inhibited growth and reduced tumor weight. In addition, the nutraceuticals induced protective epigenetic changes and increased cell death by increasing caspase gene expression. Treviso et al. (Contribution 4) evaluated the synergistic effects of the combination between the nutraceutical diallyl disulfide, an organosulfur garlic extract, and the chemotherapeutic agent 5-fluorouracil in human colorectal cancer cells (HT-29 and Caco-2). The results showed induction of apoptosis, increased genotoxicity, alteration of the global DNA methylation profile, and inhibition of cell migration, with evident differences in responses between the cell lines.

By-products resulting from food processing were also investigated. Barbosa et al. (Contribution 5) investigated the potential of agro-industrial by-products by evaluating the effects of a mango bagasse- and peel-based confectionery on gut microbiota composition, short-chain fatty acids, and hepatic gene expression in Wistar rats fed a standard diet or a high-fat diet. The results showed downregulation of genes related to lipid metabolism linked to PPAR, indicating that supplementation with functional ingredients promotes health.

Four articles addressed the role of genetic polymorphisms in different populations, focusing on aspects such as obesity, metabolic diseases, and food preferences. Gorini and Tonacci (Contribution 6) compiled articles for a current review on the relationship be-tween carbohydrate–gene interactions and the incidence and complexity of type 2 diabetes. The results showed the importance of identifying genetic polymorphisms as a potential biomarker of individual risk, with the proposal of novel foods for the prevention of the development of type 2 diabetes and the well-being of patients. Brown et al. (Contribution 7) conducted an exploratory study to investigate how genetic variation contributes to inter-individual differences in the regulation of eating behaviors. The FTO variant rs9939609 was significantly associated with higher emotional eating scores, while the CLOCK variant rs1801260 showed a nominal association with reduced postprandial craving suppression. Kim et al. (Contribution 8) conducted a study in healthy Korean adults using anthropometric analyses, clinical and genotypic assessment, and daily intake to determine the associations between genotypes and phenotypes related to obesity. The authors found significant interactions between genotype and obesity. The results showed evidence that the rs6265 variant of the BDFN gene is relevant for Koreans and highlight the importance of interpreting personalized studies in genotype-based nutrition strategies. Kim and Choi (Contribution 9) investigated whether genetic factors can influence food preferences and nutrient intake. The study involved the TAS1R2 rs35874116 gene polymorphism and diet quality in Koreans using the Korean Healthy Eating Index (KHEI). The authors concluded that the total KHEI scores did not differ between genotypes, although TT genotype carriers had significantly higher vegetable intake scores compared to C allele carriers, showing that genetic variations in sweet taste perception influence dietary behaviors among Koreans.

The articles and reviews published in this special issue of Nutrients provide readers with current new information and innovative research in elucidating the growing interest in gene-diet interactions.
